# The impact of department chair leadership identity on faculty thriving in comprehensive university reform: the cross-level moderating role of work arrangement flexibility

**DOI:** 10.3389/fpsyg.2025.1722813

**Published:** 2026-01-07

**Authors:** Jinan Gou, Yuzhao Shen

**Affiliations:** 1 School of Humanities Social Sciences (School of Public Administration), Beihang University, Beijing, China; 2 School of Management (School of Public Administration), Lanzhou University, Lanzhou, China

**Keywords:** leadership identity, faculty thriving, psychological empowerment, work arrangement flexibility, higher education reform, multilevel modeling

## Abstract

**Introduction:**

As universities undergo intensifying reform pressures, department chairs face the challenge of balancing administrative duties with academic mentorship; how they internalize their leadership role—their leadership identity—may critically shape faculty outcomes, yet this internal driver remains underexplored.

**Methods:**

Using a multi-source, two-stage design, paired data were collected from 60 department chairs and 250 faculty members across 10–14 universities undergoing “Double First-Class” reform.

**Results:**

Hierarchical linear modeling results reveal that transformational leadership significantly shapes leadership identity, which in turn exerts a positive cross-level effect on faculty thriving. Psychological empowerment partially mediates this relationship. Furthermore, work arrangement flexibility significantly moderates the leadership identity–psychological empowerment relationship, with stronger positive effects under high-flexibility conditions.

**Discussion:**

This study makes three contributions: (1) shifting leadership research focus from overt behavior to leaders’ self-concept as a driver of effectiveness; (2) enriching social information processing theory by demonstrating how internal identity becomes a perceivable cross-level social cue; and (3) providing practical implications for universities to cultivate department chairs’ leadership identity and implement flexible work policies that enhance faculty wellbeing during organizational transformation.

## 1 Introduction

Innovation and reform in higher education institutions serve as core drivers of their long-term sustainable development. In China, the “Double First-Class” initiative has intensified reform pressures, requiring universities to enhance research output while maintaining teaching quality. Faculty thriving—characterized by vitality and continuous learning—is crucial for organizational resilience and innovation (Porath et al., 2012). Identifying key factors that promote faculty thriving has therefore become a focal point in both theoretical and practical domains.

Leadership research has established that leaders significantly shape employee outcomes. Recent studies indicate that leadership style drives employee wellbeing and performance: Zhang et al. (2025) demonstrated that leader affiliative humor influences employee innovative behavior through role breadth self-efficacy and team trust; José et al. (2025) revealed that work regime moderates the relationship between leadership and quality of life. However, compared to leadership behavior, leadership identity—how leaders perceive themselves in their roles—shows greater potential for understanding leadership effectiveness (Lord and Hall, 2005). According to identity theory (Stryker and Burke, 2000; Lord and Hall, 2005), leadership identity represents a leader’s internalized self-concept, which is particularly relevant in higher education where department chairs face role ambiguity—balancing academic research with administrative responsibilities.

We draw on social information processing (SIP) theory (Salancik and Pfeffer, 1978) to explain how leadership identity influences faculty outcomes. SIP theory emphasizes that employees perceive and interpret social cues from leaders to construct their attitudes and behaviors. We extend this logic by proposing that leadership identity—though an internal self-concept—becomes a perceivable social cue through leaders’ communication, decision-making patterns, and role modeling, thereby influencing faculty psychological empowerment and thriving. Furthermore, work arrangement flexibility may shape how faculty interpret these identity cues.

Despite theoretical importance, three research gaps remain. First, while recent studies examine leadership behavior’s effects (Zhang et al., 2025; José et al., 2025), research on leadership identity as a predictor of faculty thriving and its multilevel mechanisms remains insufficient. Second, the pathway through which leadership identity affects faculty thriving via psychological empowerment has not been validated. Third, the boundary conditions—particularly how work flexibility moderates the leadership identity–empowerment relationship—remain unexplored.

Our study makes three contributions. First, responding to calls to investigate leaders’ self-concepts (Day and Sin, 2011), we shift focus from leadership behavior to leadership identity, examining its cross-level impact on faculty thriving. Second, we enrich SIP theory by demonstrating how internal identity becomes an external social cue shaping faculty empowerment. Third, by introducing work arrangement flexibility as a moderator, we reveal boundary conditions in the leadership identity–faculty outcomes relationship.

## 2 Literature and hypotheses development

### 2.1 Leadership identity and faculty thriving

Leadership identity refers to a leader’s self-concept regarding their leadership role, including beliefs about their responsibilities, capabilities, and importance (Lord and Hall, 2005). In the context of higher education reform, we define leadership identity as department chairs’ internalization of their role as change agents—that is, they view themselves as central figures in driving academic excellence and supporting faculty development. This identity encompasses three dimensions: role internalization (viewing oneself as an academic mentor rather than merely an administrative manager), responsibility awareness (recognizing one’s impact on faculty growth and organizational performance), and capability beliefs (confidence in one’s ability to lead the team through reform challenges). Unlike leader self-efficacy (which focuses only on task-specific confidence), leadership identity encompasses a broader self-schema, integrating role expectations, values, and aspirations.

Faculty thriving stems from Spreitzer et al. (2005) construct and comprises two core dimensions: vitality (energy and enthusiasm for academic work) and learning (continuously acquiring knowledge and skills). Thriving differs from job satisfaction (evaluative) or work engagement (behavioral), instead capturing a growth-oriented psychological state that enables faculty to feel both energized and continuously learning under reform pressures. Recent research indicates that systematic interventions can effectively enhance employee work engagement and thriving (Knight et al., 2017), providing a theoretical basis for organizations to promote faculty thriving through leadership development. In the context of university reform, thriving is crucial because it enables faculty to maintain wellbeing while adapting to constantly changing requirements (Porath et al., 2012).

According to SIP theory, subordinates construct attitudes and behaviors by perceiving and processing social cues from leader behavior (Salancik and Pfeffer, 1978). Unlike rational models that assume only objective information processing, SIP theory emphasizes that context shapes perception. In organizational settings, leaders serve as primary sources of social information—through their behavior, communication, and role modeling—which employees utilize to understand their work environment. We extend this logic by proposing that leadership identity—though initially an individual-level construct internal to leaders—becomes a team-level social cue when: leaders convey their self-concept through vision statements, decision rationales, and informal interactions; faculty members collectively perceive and interpret these identity signals; these perceptions shape faculty members’ psychological states and outcomes.

Transformational leadership—characterized by idealized influence, inspirational motivation, intellectual stimulation, and individualized consideration (Bass, 1985)—is thought to shape leadership identity through behavioral reinforcement and social feedback. Empirical evidence supports this relationship. Avolio et al. (2009) found that leaders who practice transformational behaviors develop stronger leadership identity over time. In higher education contexts, transformational department chairs who successfully inspire faculty during reform may internalize this success as part of their identity. Furthermore, social learning theory (Bandura, 1977) suggests that leaders shape their behavior according to organizational expectations. Universities undergoing reform signal that transformational leadership is valued (through training programs, promotion criteria). Department chairs who adopt these behaviors gradually internalize a “reform leader” identity. Therefore, we propose:

*H1*: Department chairs’ transformational leadership style (rated by faculty, L2) has a significant positive effect on their leadership identity (L2).

SIP theory suggests that leadership identity, as a team-level social cue, is collectively perceived and interpreted by faculty, thereby producing cross-level effects. Leadership identity becomes a perceivable team-level cue through chairs’ communication, behavioral consistency, and symbolic actions (e.g., advocating for faculty resources), which faculty collectively observe and interpret. Similar cross-level effects have received empirical support (Edmondson, 1999; José et al., 2025). We extend this by arguing that leaders’ self-concepts (not just behaviors) are also observable and influential. Therefore, we hypothesize:

*H2*: Department chairs’ leadership identity (L2) has a significant positive cross-level effect on faculty thriving (L1).

### 2.2 The mediating role of psychological empowerment

Psychological empowerment—comprising meaning, competence, self-determination, and impact (Spreitzer, 1995)—is a crucial cognitive state for faculty in reform contexts. Empowered faculty feel their work is meaningful, believe in their capabilities, exercise autonomy, and perceive impact on departmental outcomes. We distinguish empowerment from role breadth self-efficacy (studied by Zhang et al., 2025). While self-efficacy focuses on capability beliefs, empowerment encompasses perceived autonomy and meaning—dimensions particularly salient in academia where intellectual freedom is paramount.

Psychological empowerment is particularly appropriate as a mediator in academic contexts for three reasons. First, unlike corporate settings where formal authority dominates, academic work relies heavily on intrinsic motivation; empowerment’s meaning and self-determination dimensions capture faculty’s need for intellectual purpose and autonomy. Second, empowerment encompasses perceived impact—faculty’s belief that their work matters to the department—which is critical in reform contexts where roles may feel uncertain. Third, compared to alternative mediators such as job satisfaction (evaluative) or self-efficacy (capability-focused), empowerment more comprehensively captures the cognitive appraisal process through which faculty interpret leadership signals and translate them into thriving.

SIP theory suggests that leaders’ identity signals shape employees’ self-perceptions (Salancik and Pfeffer, 1978). Department chairs with strong leadership identity are more likely to: delegate decision-making—leaders who view themselves as “mentors” trust faculty to make decisions, enhancing self-determination; articulate meaning—leaders who identify with the reform mission communicate how faculty work contributes to organizational goals, elevating perceived meaning; provide resources—leaders confident in their identity advocate for funding and support for faculty projects, enhancing competence perception. Recent research supports this. Hermanto et al. (2024) found that transformational leadership enhances psychological empowerment. Similarly, Wang et al. (2022) confirmed that high-involvement work practices promote employee innovative behavior through psychological empowerment, indicating that empowerment plays a crucial mediating role between leadership effectiveness and employee outcomes. Xu et al. (2022) also found that self-sacrificial leadership promotes organizational learning by inspiring work passion, further supporting the impact of leadership style on employee psychological states. We extend this by arguing that leadership identity (not just behavior) drives empowerment—because identity represents a more stable, internalized driver of supportive actions. Therefore, we hypothesize:

*H3*: Department chairs’ leadership identity (L2) has a significant positive effect on faculty psychological empowerment (L1).

Empowered faculty are more likely to thrive because: autonomy promotes learning—self-determination (a core dimension of empowerment) enables faculty to pursue intellectual interests, driving continuous learning (Deci and Ryan, 2000); meaning enhances vitality—when faculty perceive their work as meaningful, they experience greater passion and energy (Spreitzer et al., 2005); resource availability—empowerment signals that resources are available, reducing stress and allowing cognitive resources to focus on growth. Empirical evidence is strong. Ngo et al. (2023) found that empowerment predicts proactive behavior. Porath et al. (2012) demonstrated that empowerment fosters thriving in organizational contexts. We extend this to academia. Combining H2, H3, and H4, we propose a cross-level mediation model: leadership identity (L2) → psychological empowerment (L1) → faculty thriving (L1). This aligns with multilevel mediation logic (Preacher et al., 2010) and SIP theory’s emphasis on cognitive mechanisms. Therefore, we hypothesize:

*H4*: Faculty psychological empowerment (L1, T1) has a significant positive effect on their thriving (L1, T2).

### 2.3 The moderating role of work arrangement flexibility

We define work arrangement flexibility as faculty’s perceived autonomy over when, where, and how to conduct academic work (Mache et al., 2020).

SIP theory suggests that contextual factors shape how individuals process social cues (Salancik and Pfeffer, 1978). According to situational strength theory (Meyer et al., 2010), situational characteristics either enhance or diminish relationships between individual traits and behaviors. In this study, work arrangement flexibility, as an important contextual factor, may moderate the impact of leadership identity. We propose that work arrangement flexibility moderates the leadership identity → psychological empowerment relationship through cue salience. In high-flexibility contexts, when departments provide high flexibility, faculty have greater autonomy—making leader support a key determinant of how they utilize this freedom. In this case, the leader’s identity as a “mentor” becomes highly salient: faculty closely attend to whether leaders genuinely support their autonomous work. If leaders exhibit strong identity (e.g., consistently advocating for faculty interests), faculty interpret flexibility as empowerment (“I have freedom, and my leader supports me”). This amplifies empowerment. In low-flexibility contexts, when flexibility is low (rigid schedules, mandatory office hours), faculty empowerment depends more on structural constraints than leadership identity. Even if leaders have strong identity, limited flexibility restricts actual autonomy, weakening the leadership identity-empowerment link.

This logic differs from José et al. (2025), who found that work regime moderates leadership effects on quality of life. We focus on empowerment as an outcome, arguing that flexibility’s moderating role is strongest for this cognitive state. Similar moderating effects have been documented. Huang and Luthans (2015) argue that individual behavior requires examining person-environment interactions. De Jong et al. (2016) found that team climate moderates leadership effects. We extend this by showing that work flexibility (a policy-driven contextual factor) shapes leadership’s psychological impact. We propose that work flexibility moderates this relationship through cue salience: in high-flexibility contexts, faculty possess autonomy but face uncertainty, making leadership identity signals more salient as guides for action; in low-flexibility contexts, structural constraints dominate, reducing leaders’ relative influence (Meyer et al., 2010). Therefore, we hypothesize:

*H5*: Work arrangement flexibility (L2) moderates the relationship between leadership identity (L2) and psychological empowerment (L1), such that when flexibility is higher, the positive relationship is stronger.

**FIGURE 1:**
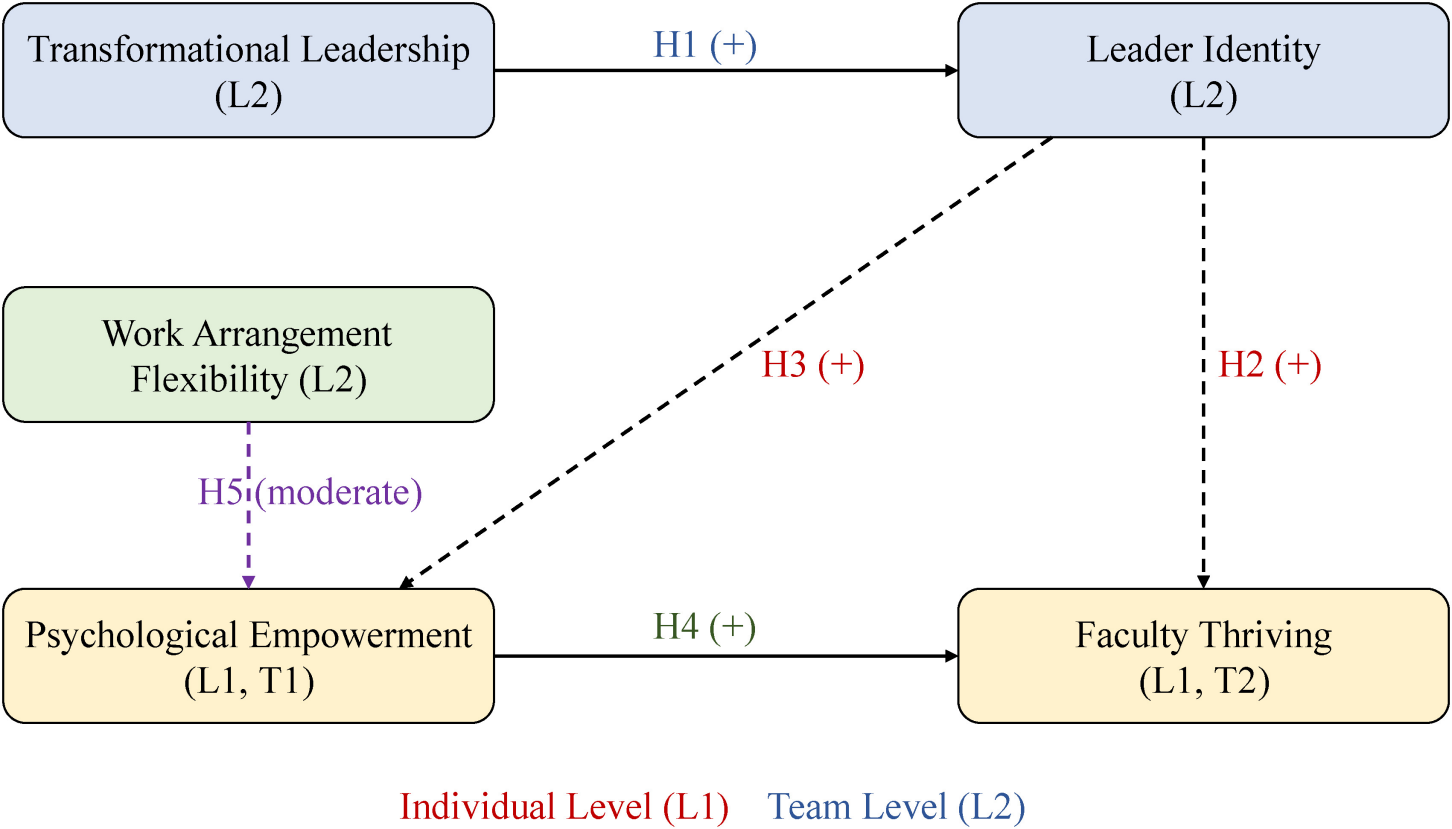
Research conceptual model.

Figure 1 summarizes our conceptual model, depicting the five hypothesized relationships.

## 3 Materials and methods

### 3.1 Sample and procedure

This study sampled department chairs and faculty from universities in China undergoing “Double First-Class” reform. We recruited participants from 10 to 14 universities using snowball sampling. Snowball sampling was adopted because comprehensive lists of department chairs undergoing reform are not publicly available. To mitigate potential selection bias, we initiated recruitment from multiple independent starting points across different regions and set quotas to ensure representation across disciplines (STEM, social sciences, humanities). All participants in this study provided informed consent before participation.

To reduce common method bias (Podsakoff et al., 2012), this study adopted a multi-source, two-stage longitudinal design. Data collection was conducted from April to October 2024. Time 1 (T1, April-June 2024): Faculty completed questionnaires rating department chairs’ transformational leadership, their own psychological empowerment, work arrangement flexibility, and demographic information. Simultaneously, department chairs completed questionnaires self-rating leadership identity, reform intensity, and demographic information. Time 2 (T2, September-October 2024, 3-month interval): Department chairs rated each participating faculty member’s thriving performance over the past 3 months. The 3-month interval was determined based on theoretical and practical considerations: sufficient time for psychological empowerment to manifest in observable thriving behaviors (Spreitzer et al., 2005), alignment with the academic semester structure, and methodological recommendations for reducing common method bias while minimizing attrition (Podsakoff et al., 2012). After removing invalid questionnaires and unmatched samples, we ultimately obtained valid paired data from 60 department chairs and 250 faculty members, with an effective questionnaire recovery rate of 57.1%.

According to multilevel modeling sample size requirements (Maas and Hox, 2005), the number of level-2 units should be at least 30 to obtain reliable estimates. This study’s nested data of 60 department chairs and 250 faculty members satisfies the sample size requirements for multilevel analysis. Furthermore, based on Cohen’s (1988) statistical power analysis, this sample size is sufficient to detect cross-level effects with medium effect size (*d* = 0.50), achieving statistical power above 0.80.

Department chair sample (*n* = 60): Males accounted for 65%, females 35%; age *M* = 48.3 years (SD = 7.2); professors 50%, associate professors 40%; average tenure as department chair *M* = 5.7 years (SD = 3.1). Faculty sample (*n* = 250): Males 58%, females 42%; age *M* = 36.8 years (SD = 8.5); associate professors 50%, professors 30%, assistant professors 15%; average years in current department *M* = 6.2 years (SD = 4.8); doctoral degree holders 85%. Average number of faculty per department/division was 4.2 (range: 3–8).

### 3.2 Measurement instruments

All measurement scales employed in this study are sourced from validated established scales and adopted a back-translation procedure to ensure cross-cultural validity. Unless otherwise specified, all scales used a 5-point Likert scale (1 = strongly disagree, 5 = strongly agree).

#### 3.2.1 Transformational leadership (L2, rated by faculty at T1)

The 20-item version of Bass (1985) Multifactor Leadership Questionnaire (MLQ-5X) was adopted, assessing five dimensions: idealized influence, inspirational motivation, intellectual stimulation, individualized consideration, and change-promoting behavior. Sample item: “My department chair clearly communicates a vision for our department/program development.” Aggregation statistics: rwg = 0.89, ICC (1) = 0.21, ICC (2) = 0.82, supporting aggregation to L2 (James et al., 1993) Cronbach’s α = 0.94.

#### 3.2.2 Leadership identity (L2, department chair self-rating at T1)

Adapted from Lord and Hall (2005) 8-item scale, assessing role internalization, responsibility awareness, and capability beliefs. Sample item: “I consider ‘academic leader’ to be a core part of my self-concept” Cronbach’s α = 0.91.

#### 3.2.3 Psychological empowerment (L1, faculty self-rating at T1)

Spreitzer (1995) 12-item scale was adopted, assessing four dimensions: meaning, competence, autonomy, and impact. Sample item: “I can autonomously decide on research directions and topics” Cronbach’s α = 0.88.

#### 3.2.4 Work arrangement flexibility (L2, rated by faculty at T1 and aggregated)

Adapted from Mache et al.’s (2020) 5-item scale, assessing temporal, spatial, and task autonomy. Sample item: “I can flexibly arrange my work location during non-teaching hours.” Aggregation statistics: rwg = 0.87, ICC (1) = 0.19, ICC (2) = 0.80 Cronbach’s α = 0.85.

#### 3.2.5 Faculty thriving (L1, rated by department chairs at T2)

Adapted from Porath et al. (2012) 10-item scale, comprising two dimensions: vitality (5 items) and learning (5 items). Sample item: “This faculty member demonstrates abundant energy in teaching and research work” (vitality). To avoid common method bias, department chair ratings were adopted (Podsakoff et al., 2012) Cronbach’s α = 0.90.

Control Variables. At the individual level (L1), we controlled for gender, age, professional rank, and years in department. At the team level (L2), we controlled for reform intensity (3 items, Cronbach’s α = 0.82), team size, and discipline category.

### 3.3 Data analysis strategy

We used hierarchical linear modeling (HLM 6.08) to handle the nested data structure. Analysis proceeded stepwise: (1) confirmatory factor analysis (CFA) using AMOS 22.0 to examine discriminant validity of constructs; (2) Harman’s single-factor test and VIF analysis to assess common method bias; (3) calculation of rwg, ICC (1), ICC (2) to verify aggregation rationality of L2 variables; (4) calculation of descriptive statistics and correlation coefficients.

For hypothesis testing, H1 employed team-level simple regression. H2 and H3 used cross-level main effects models to test L2 variables’ effects on L1 outcomes. H4 used within-level effects models to test psychological empowerment’s (L1, T1) effect on thriving (L1, T2). H5 used cross-level moderation models, with interaction terms testing work flexibility’s moderating effect, and simple slope analysis at ± 1 SD flexibility levels. Additionally, Bootstrap methods (5,000 iterations) were used to calculate confidence intervals for mediation effects.

## 4 Results

### 4.1 Confirmatory factor analysis

We conducted confirmatory factor analysis (CFA) using AMOS 22.0 at the individual level to examine discriminant validity. For variables aggregated to Level 2 (transformational leadership, work flexibility), individual-level CFA is appropriate because: (1) these constructs are measured at the individual level before aggregation; (2) our Level-2 sample size (*n* = 60) is insufficient for stable team-level CFA (recommended *n* > 200; Kline, 2015); (3) aggregation validity is separately established through rwg, ICC (1), and ICC (2) indices reported in the Measurement section, which confirm sufficient within-group agreement and between-group variance to justify aggregation (James et al., 1993). This approach is consistent with multilevel research practices (Chen et al., 2004). We compared the hypothesized five-factor model (transformational leadership, leadership identity, psychological empowerment, work flexibility, faculty thriving) with several alternative models. As shown in Table 1, the hypothesized five-factor model exhibited good fit indices: χ^2^/df = 1.78, CFI = 0.96, TLI = 0.95, GFI = 0.91, RMSEA = 0.054, SRMR = 0.048. In contrast, the four-factor model (combining leadership identity with transformational leadership), three-factor model (combining psychological empowerment with thriving), and single-factor model all showed significantly worse fit indices (all Δχ^2^ tests, *p* < 0.001). These results support discriminant validity among the five constructs.

**TABLE 1:** Confirmatory factor analysis results.

Model	χ^2^/df	CFI	TLI	GFI	RMSEA	SRMR	Δ χ^2^
Hypothesized five-factor model	1.78	0.96	0.95	0.91	0.054	0.048	–
Four-factor model^a^	3.45	0.88	0.86	0.82	0.092	0.105	312.5***
Three-factor model^b^	4.62	0.81	0.78	0.76	0.118	0.135	489.2***
Single-factor model^c^	7.89	0.58	0.54	0.61	0.165	0.198	1,024.8***

*N* = 250 (individual level). The five-factor model includes transformational leadership, leadership identity, psychological empowerment, work flexibility, and faculty thriving.

^a^Four-factor model: combining transformational leadership with leadership identity.

^b^Three-factor model: further combining psychological empowerment with faculty thriving based on the four-factor model.

^c^Single-factor model: all variables combined into one factor. Δχ^2^ = chi-square difference relative to the hypothesized five-factor model.

***p < 0.001.

Additionally, we assessed convergent and discriminant validity. As shown in Table 1, all standardized factor loadings exceeded 0.60, composite reliability (CR) values ranged from 0.85 to 0.94, and average variance extracted (AVE) values ranged from 0.52 to 0.61, all exceeding the recommended thresholds (Hair et al., 2009). For discriminant validity, the square root of each construct’s AVE exceeded its correlations with other constructs, satisfying the Fornell-Larcker criterion.

### 4.2 Common method bias

Given that transformational leadership, psychological empowerment, and work flexibility were all rated by faculty at T1, we employed multiple methods to assess common method bias. First, Harman’s single-factor test showed that the first factor in unrotated exploratory factor analysis explained 28.4% of variance, below the recommended 50% threshold. Second, collinearity analysis revealed that variance inflation factors (VIF) for all variables were below 2.5, and tolerance exceeded 0.40, indicating no severe multicollinearity problems. Additionally, procedural remedies we adopted—multi-source data (department chair ratings of faculty thriving) and temporal separation (3-month T1-T2 interval)—further reduced common method bias risk.

### 4.3 Descriptive statistics and correlation analysis

As shown in Table 2, we report means, standard deviations, CR, AVE, and correlation coefficients for each variable. All CR values exceeded 0.85 and AVE values exceeded 0.50, indicating adequate convergent validity (Hair et al., 2009). Transformational leadership (*M* = 3.68, SD = 0.72), leadership identity (*M* = 3.82, SD = 0.65), psychological empowerment (*M* = 3.45, SD = 0.81), work flexibility (*M* = 3.51, SD = 0.68), and faculty thriving (*M* = 3.76, SD = 0.74) all had means above the scale midpoint (3.0), indicating the sample overall was at medium-to-high levels. All scales’ Cronbach’s α coefficients exceeded 0.85 (range: 0.85–0.94), indicating good internal consistency.

**TABLE 2:** Means, standard deviations, reliability, validity, and correlations.

Variable	M	SD	CR	AVE	1	2	3	4	5
1. Transformational Leadership (L2)	3.68	0.72	0.94	0.54	(0.94)				
2. Leadership identity (L2)	3.82	0.65	0.91	0.58	0.54***	(0.91)			
3. Psychological empowerment (L1)	3.45	0.81	0.88	0.55	0.38***	0.42***	(0.88)		
4. Work flexibility (L2)	3.51	0.68	0.85	0.52	0.29**	0.35**	0.41***	(0.85)	
5. Faculty thriving (L1)	3.76	0.74	0.90	0.61	0.46***	0.51***	0.55***	0.39***	(0.90)

*N* = 250 (individual level, L1), 60 (team level, L2). Values in parentheses are Cronbach’s α coefficients. CR, composite reliability; AVE, average variance extracted. The square root of AVE for each construct (0.73, 0.76, 0.74, 0.72, 0.78) exceeds inter-construct correlations, supporting discriminant validity. L1 = individual level; L2 = team level. Correlations involving L2 variables (TL, LI, WF) are based on aggregated team-level scores and are reported for descriptive purposes only; all inferential analyses rely on hierarchical linear modeling (HLM). **p < 0.05, ***p < 0.01, ****p < 0.001.

Correlation analysis showed all variables were significantly positively correlated. Specifically, leadership identity was significantly positively correlated with faculty thriving (*r* = 0.51, *p* < 0.001), providing preliminary support for H2. Psychological empowerment was also significantly positively correlated with faculty thriving (*r* = 0.55, *p* < 0.001), providing preliminary evidence for H4. Furthermore, the correlation coefficient between transformational leadership and leadership identity was 0.54 (*p* < 0.001), and between work flexibility and psychological empowerment was 0.41 (*p* < 0.001), with all correlation directions consistent with hypotheses.

### 4.4 Hypothesis testing

*H1*: Effect of transformational leadership on leadership identity. We conducted simple regression analysis at the team level (*n* = 60) to test H1. As shown in Table 3, transformational leadership had a significant positive effect on leadership identity (β = 0.64, SE = 0.12, *t* = 5.33, *p* < 0.001), and this model explained 29% of variance in leadership identity (*R*^2^ = 0.29). Therefore, H1 was supported.

**TABLE 3:** Regression analysis results of transformational leadership on leadership identity.

Predictor variable	B	SE	β	*t*	*p*
Constant	1.23	0.28	–	4.39	< 0.001
**Main effect**					
Transformational leadership	0.64	0.12	0.54	5.33	< 0.001
**Control variables**					
Chair age	−0.01	0.01	−0.08	−1.12	0.27
Chair gender^a^	0.08	0.12	0.05	0.67	0.51
Reform intensity	0.15	0.09	0.13	1.67	0.10
Tenure as chair	0.02	0.02	0.07	1.00	0.32
*R* ^2^	0.29				
*F*	5.89***				
df	(5, 54)				

*N* = 60 (team level). Dependent variable: leadership identity.

^a^Gender coding: 0 = male, 1 = female.

***p < 0.001.

*H2*: Cross-level main effect of leadership identity on faculty thriving. We first established a null model to calculate intraclass correlation coefficients (ICC). Results showed between-group variance in faculty thriving was 0.15, within-group variance was 0.38, ICC (1) = 0.28, indicating 28% of thriving variance exists between teams, demonstrating the necessity of HLM. Subsequently, we tested leadership identity’s cross-level main effect. Following Raudenbush and Bryk (2002), we specified random intercept models with fixed slopes. Random slope models were tested for robustness; results showed non-significant slope variance (τ _11_ = 0.02, *p* = 0.18), supporting the fixed slope specification. As shown in Table 4, after controlling for reform intensity, team size, and individual-level gender, age, and professional rank, leadership identity had a significant positive effect on faculty thriving (γ = 0.48, SE = 0.09, *t* = 5.33, *p* < 0.001). Therefore, H2 was supported.

**TABLE 4:** Cross-level effects of leadership identity on faculty thriving and mediating role of psychological empowerment.

Variable	Model 1	Model 2	Model 3	Model
	Null model	H2: LI→thriving	H3: LI→empowerment	Mediation model
**Fixed effects**				
Intercept (γ_00_)	3.76*** (0.07)	3.76*** (0.08)	3.45*** (0.08)	3.76*** (0.07)
**L2 predictors**				
Leadership identity (γ_01_)	–	0.48*** (0.09)	0.52*** (0.10)	0.28** (0.09)
Reform intensity (γ_02_)	–	−0.12 (0.07)	−0.08 (0.08)	−0.10 (0.06)
Team size (γ_03_)	–	0.03 (0.05)	0.02 (0.06)	0.02 (0.04)
**L1 predictors**				
Psychological empowerment (γ_10_)	–	–	–	0.38*** (0.06)
Gender (γ_11_)	–	0.05 (0.07)	0.04 (0.08)	0.03 (0.06)
Age (γ_12_)	–	−0.01 (0.01)	0.00 (0.01)	−0.00 (0.01)
Professional rank (γ_13_)	–	0.08 (0.06)	0.06 (0.07)	0.06 (0.05)
**Variance components**				
L1 residual (σ^2^)	0.38	0.32	0.36	0.25
L2 intercept (τ_00_)	0.15	0.08	0.11	0.06
ICC (1)	0.28	–	–	–
**Model fit**				
Deviance	682.4	651.2	668.5	632.8
ΔDeviance (vs. M1)	–	31.2***	13.9**	49.6***

*N* = 250 (individual level, L1), 60 (team level, L2). Dependent variable: models 1–2 and model 4 are faculty thriving; model 3 is psychological empowerment. Values in parentheses are standard errors. LI, Leadership Identity. **p < 0.01, ***p < 0.001.

*H3 and H4*: Mediating role of psychological empowerment. To test psychological empowerment’s mediating effect, we followed Mathieu and Taylor’s (2007) multilevel mediation procedure. Our model follows a 2-1-1 structure (Preacher et al., 2010): leadership identity (L2) → psychological empowerment (L1) → faculty thriving (L1). This specification is appropriate because leadership identity represents a shared team-level cue, while empowerment and thriving vary at the individual level as faculty differentially respond to leader signals. First, as shown in Table 4, leadership identity had a significant positive effect on psychological empowerment (γ = 0.52, SE = 0.10, *t* = 5.20, *p* < 0.001), supporting H3. Second, after controlling for leadership identity, psychological empowerment had a significant positive effect on faculty thriving (γ = 0.38, SE = 0.06, *t* = 6.33, *p* < 0.001), supporting H4. When both leadership identity and psychological empowerment were entered into the model, leadership identity’s direct effect on faculty thriving weakened but remained significant (γ = 0.28, SE = 0.09, *p* < 0.01), indicating psychological empowerment plays a partial mediating role. The indirect effect calculated using Bootstrap methods (5,000 iterations) was 0.20 [95% CI (0.14, 0.28)], with confidence intervals not including zero, further confirming significant mediation. Therefore, H3 and H4 were both supported.

**FIGURE 2:**
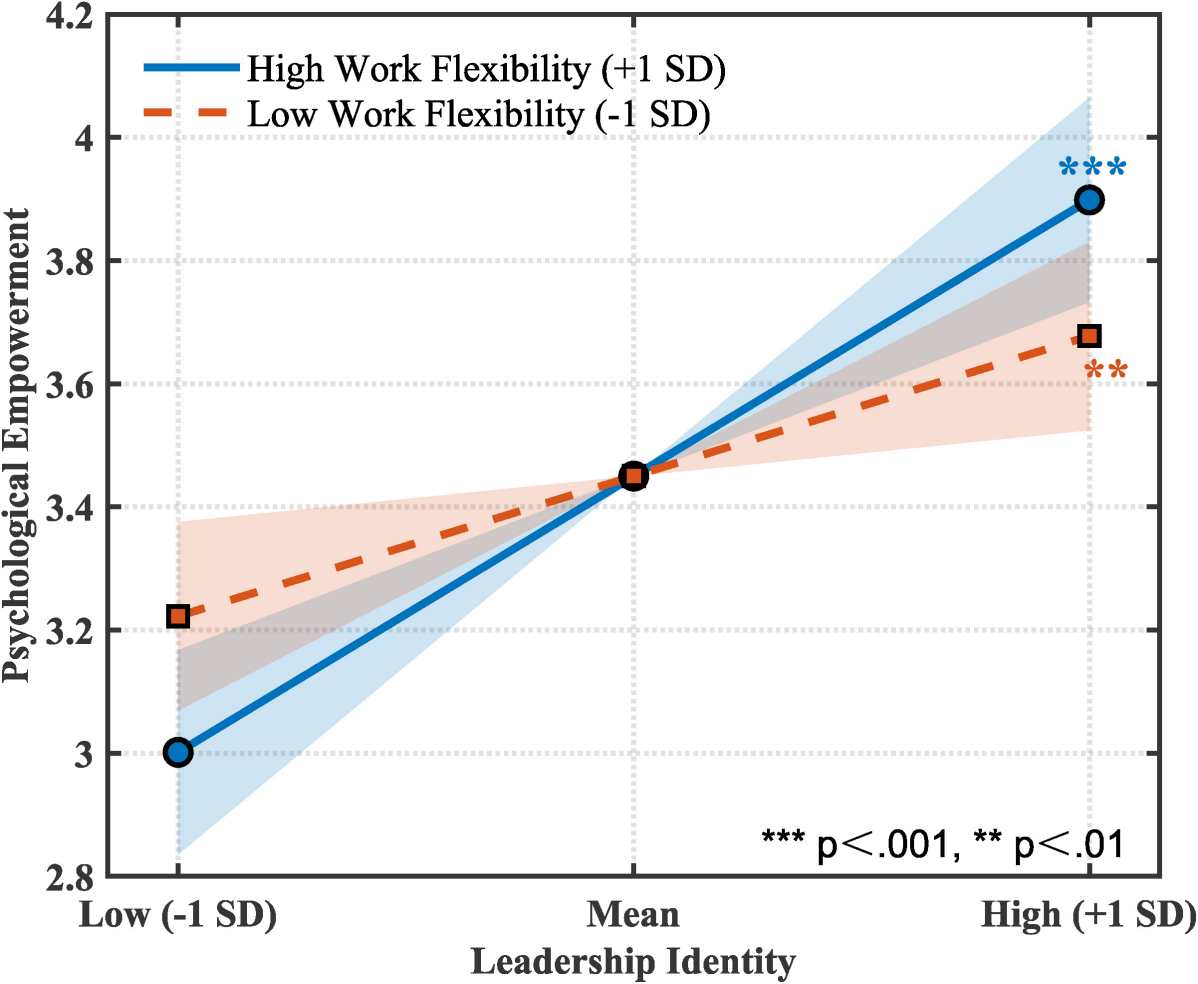
Moderating effect of work flexibility. Shaded areas represent 95% confidence intervals for simple slopes.

*H5*: Cross-level moderating role of work flexibility. To test work flexibility’s moderating effect on the leadership identity-psychological empowerment relationship, we established a cross-level model including interaction terms. As shown in Table 5, the interaction between leadership identity and work flexibility had a significant positive effect on psychological empowerment (γ = 0.24, SE = 0.11, *t* = 2.18, *p* = 0.03), and this interaction term explained an additional 5% of variance (Δ*R*^2^ = 0.05). To further interpret this interaction effect, we conducted simple slope analysis at high flexibility (+ 1 SD) and low flexibility (−1 SD) conditions. Results showed that under high flexibility conditions, leadership identity’s effect on psychological empowerment was stronger (simple slope = 0.69, SE = 0.13, *t* = 5.31, *p* < 0.001), while under low flexibility conditions, this effect was relatively weaker (simple slope = 0.35, SE = 0.12, *t* = 2.92, *p* = 0.005). As shown in Figure 2, the interaction effect graph clearly demonstrates this moderating pattern: when work flexibility is higher, leadership identity’s positive effect on psychological empowerment is more significant. Therefore, H5 was supported.

**TABLE 5:** Cross-level moderating effect of work flexibility on leadership identity-psychological empowerment relationship.

Predictor variable	γ	SE	*t*	*p*	95% CI
**Main effects model**					
Intercept (γ_00_)	3.45***	0.07	49.29	< 0.001	(3.31, 3.59)
Leadership identity (γ_01_)	0.52***	0.10	5.20	< 0.001	(0.32, 0.72)
Work flexibility (γ_02_)	0.31**	0.09	3.44	0.001	(0.13, 0.49)
**Moderation model**					
Intercept (γ_00_)	3.45***	0.07	49.29	< 0.001	(3.31, 3.59)
Leadership identity (γ_01_)	0.52***	0.10	5.20	< 0.001	(0.32, 0.72)
Work flexibility (γ_02_)	0.31**	0.09	3.44	0.001	(0.13, 0.49)
Identity × flexibility (γ_03_)	0.24*	0.11	2.18	0.03	(0.02, 0.46)
**Simple slope analysis**					
High work flexibility (+ 1 SD)	0.69***	0.13	5.31	< 0.001	(0.43, 0.95)
Low work flexibility (−1 SD)	0.35**	0.12	2.92	0.005	(0.11, 0.59)
Slope difference	0.34*	0.16	2.13	0.04	(0.02, 0.66)
**Variance components**					
L1 residual (σ^2^)	0.33				
L2 intercept (τ_00_)	0.09				
**Model comparison**					
ΔR^2^ (Interaction contribution)	0.05				
ΔDeviance	4.75*				

*N* = 250 (individual level), 60 (team level). Dependent variable: psychological empowerment. CI, confidence interval. *p < 0.05, **p < 0.01, ***p < 0.001.

As shown in Table 6, we summarized all hypothesis testing results. All five hypotheses received empirical support.

**TABLE 6:** Summary of hypothesis testing results.

Hypothesis	Path	Coefficient	SE	t/F	*p*	Result
H1	Transformational leadership → leadership identity (L2→L2)	β = 0.64	0.12	5.33	< 0.001	Supported^a^^,^^b^
H2	Leadership identity → faculty thriving (L2→L1)	γ = 0.48	0.09	5.33	< 0.001	Supported^a^^,^^b^
H3	Leadership identity → psychological empowerment (L2→L1)	γ = 0.52	0.10	5.20	< 0.001	Supported^a^^,^^b^
H4	Psychological empowerment → faculty thriving (L1→L1)	γ = 0.38	0.06	6.33	< 0.001	Supported^a^^,^^b^
H5	Work flexibility moderates LI→Emp (L2 × L2→L1)	γ = 0.24	0.11	2.18	0.03	Supported^a^^,^^b^

LI, Leadership Identity; Emp, Psychological Empowerment; Thriving, Faculty Thriving. L1, individual level; L2 = team level.

^a^Indirect effect based on Bootstrap method (5,000 iterations), 95% CI (0.14, 0.28).

^b^Bootstrap confidence interval does not include zero, indicating significant indirect effect.

## 5 Discussion

Based on social information processing theory, this study explored the multilevel impact mechanism of department chair leadership identity on faculty thriving. Findings revealed: (1) transformational leadership has a significant positive effect on leadership identity; (2) leadership identity has a significant positive cross-level effect on faculty thriving; (3) psychological empowerment plays a partial mediating role; (4) work arrangement flexibility positively moderates the leadership identity-psychological empowerment relationship. These findings provide important implications for leadership research, SIP theory application, and higher education management practice.

### 5.1 Theoretical contributions

This study makes three theoretical contributions. First, we respond to calls for research on leaders’ self-concepts (Day and Sin, 2011; Lord and Hall, 2005), shifting focus from leadership behavior to leadership identity. Zhang et al. (2025) studied how leader humor behavior influences employee innovation, while José et al. (2025) explored how work regimes moderate leadership effects. This study focuses on leaders’ self-concepts (who leaders think they are). We found leadership identity strongly responds to transformational leadership (β = 0.64) and produces cross-level effects on faculty thriving (γ = 0.48). This extends identity theory to higher education, revealing that leaders’ self-cognition is an internal driver of behavior.

Our findings suggest that leadership identity is transmitted to faculty through observable channels. Department chairs with strong identity communicate their role commitment verbally (e.g., articulating vision in meetings), demonstrate behavioral consistency (e.g., persistent mentoring and resource advocacy), and engage in symbolic actions (e.g., attending faculty presentations, defending departmental interests). Faculty collectively perceive these signals, interpret them as indicators of genuine leadership commitment, and consequently experience enhanced empowerment. This transmission process explains how an internal self-concept produces cross-level effects on individual faculty outcomes.

Second, this study enriches SIP theory applications. Previous research focused on leadership behavior as social cues (Zhang et al., 2025), while this study demonstrates that leadership identity—though an internal concept—can also be perceived by faculty through communication and role modeling. We found leadership identity promotes thriving through psychological empowerment (γ = 0.52) (indirect effect = 0.20). Psychological empowerment encompasses meaning, competence, autonomy, and impact, better capturing faculty needs for academic freedom than self-efficacy alone. Furthermore, work flexibility significantly moderated the leadership identity-empowerment relationship (γ = 0.24), with stronger effects under high flexibility conditions (simple slope = 0.69 vs. 0.35). This extends José et al. (2025) work regime moderation effects, revealing flexibility’s mechanism: department chairs with strong leadership identity can help faculty transform flexibility into empowerment.

The moderating effect, while statistically significant (γ = 0.24, *p* = 0.03), was modest in magnitude. Several contextual factors may explain this finding. First, academic work is characterized by inherently high baseline autonomy—faculty already possess considerable freedom in research focus and teaching methods—which may create ceiling effects limiting additional variance from formal flexibility policies. Second, our sample’s relatively small team sizes (*M* = 4.2 members) reduce observable variation in flexibility practices across departments. Third, institutional constraints in Chinese universities—such as mandatory office hours, administrative meetings, and teaching schedules—may attenuate the full expression of flexibility policies. Despite its modest size, this effect is practically meaningful: a one-standard-deviation increase in flexibility nearly doubles the leadership identity–empowerment slope (from 0.35 to 0.69), indicating that flexibility policies can meaningfully amplify leadership effectiveness in academic settings.

Third, this study provides unique insights into leadership effectiveness in loosely coupled academic organizations (Weick, 1976). Unlike corporate settings with clear hierarchies and direct supervision, universities are characterized by professional autonomy, decentralized decision-making, and weak formal authority. In such contexts, department chairs cannot rely on positional power; instead, their influence depends on whether faculty perceive them as legitimate academic leaders. Our findings demonstrate that leadership identity—chairs’ internalized self-concept as change agents—serves as a crucial mechanism for exerting cross-level influence in loosely coupled systems. When chairs communicate their identity through consistent behavior and symbolic actions, they bridge the structural gaps inherent in academic organizations, enabling their influence to reach individual faculty members despite weak formal linkages.

Furthermore, our study contributes contextualized evidence from China’s “Double First-Class” reform. This initiative creates intense pressure for research excellence while demanding administrative efficiency—placing department chairs in a unique position of role conflict. Our findings that leadership identity promotes faculty thriving (γ = 0.48) even under such pressure suggests that internalized role commitment may buffer against reform-induced stress. This evidence extends Western-developed leadership theories to collectivist cultural contexts, where role modeling and relational leadership may carry particular weight.

### 5.2 Practical implications

This study provides three practical implications for higher education management. First, universities should emphasize cultivating department chairs’ leadership identity rather than focusing solely on skills training. Specific measures include: organizing reflective workshops to guide department chairs in thinking about their leadership role positioning; establishing mentorship systems where senior chairs guide newcomers; assessing identification with academic leadership roles in selection and evaluation. This study shows transformational leadership can shape leadership identity (β = 0.64), so training should help department chairs integrate transformational leadership principles into daily practice.

Second, department chairs should consciously enhance faculty psychological empowerment. This study revealed empowerment’s mediating role (indirect effect = 0.20). Specific measures include: clearly communicating connections between departmental vision and faculty work (meaning); providing resource support and constructive feedback (competence); granting freedom in course design, research directions, and work time (autonomy); inviting faculty participation in important decisions (impact).

Third, work flexibility policies must be combined with leadership support. This study found flexibility’s moderating effect (γ = 0.24), suggesting that simply increasing flexibility is insufficient for enhancing faculty wellbeing. When implementing flexible policies, universities should simultaneously strengthen department chair development to ensure effective guidance in flexible environments. Specific recommendations include formulating flexible work guidelines, establishing virtual platforms and regular team meeting mechanisms, and recognizing chairs who effectively support faculty.

### 5.3 Limitations and future research directions

This study has limitations. First, although adopting a two-stage design, H1 measured at the same time point, limiting causal inference. Future research should adopt longer timeframe designs to track identity’s dynamic evolution. Second, leadership identity used self-rating, potentially introducing bias. Future studies could adopt multi-source data (self-rating, faculty perception, behavioral observation) for triangulation, or use qualitative research to deeply explore how department chairs construct leadership identity.

Third, this study’s sample comes from Chinese universities, limiting cultural generalizability. In collectivist cultures, leadership role modeling’s influence may be more prominent. Future research should replicate studies in different cultural contexts, examining cultural factors’ (such as power distance, individualism-collectivism) moderating effects. Fourth, this study focused on positive outcomes, not exploring leadership identity’s potential negative effects. Overly strong identity may lead to rigidity or ignoring faculty heterogeneous needs. Future research should explore potential negative effects and boundary conditions (such as leader humility), as well as faculty individual differences’ moderating roles.

Finally, this study focused on psychological empowerment as mediator, but influence pathways may be more complex. Future research could explore other mediators (such as trust, organizational identification) and moderators (such as organizational support, reform uncertainty). Additionally, relatively small sample size may limit statistical power for testing complex models. Future research should expand samples, including more universities and different types of departments, or adopt experimental designs to rigorously test causal relationships.

Future research could also explore other leadership styles’ (such as humble leadership) roles in higher education reform. Lei et al. (2022) found humble leadership promotes team innovation through team reflexivity—this leadership style may have unique advantages in academic environments. Comparing different leadership identities’ (such as change agent vs. humble servant) differential impacts on faculty outcomes will provide richer insights for leadership theory.

Additionally, our Level-2 sample size (*n* = 60 teams), while meeting minimum requirements for multilevel modeling (Maas and Hox, 2005), may limit statistical power for detecting small cross-level interaction effects and constrains our ability to test more complex random slope models or conduct team-level CFA.

## 6 Conclusion

This study examined how department chair leadership identity influences faculty thriving in Chinese higher education reform contexts. Our multilevel analysis of 60 department chairs and 250 faculty members yielded three key findings: (1) transformational leadership shapes leadership identity (β = 0.64); (2) leadership identity enhances faculty thriving through psychological empowerment (indirect effect = 0.20); (3) work arrangement flexibility amplifies the identity–empowerment relationship (γ = 0.24).

These findings carry important implications. For theory, we demonstrate that leaders’ self-concepts—not just behaviors—serve as cross-level social cues affecting employee outcomes. For practice, universities should invest in identity development programs for department chairs and ensure that flexible work policies are accompanied by leadership support.

This study opens several avenues for future research. First, longitudinal designs tracking identity evolution over multiple time points would strengthen causal inference. Second, cross-cultural replications could examine whether identity transmission mechanisms differ across cultural contexts. Third, exploring potential negative effects of overly strong leadership identity (e.g., rigidity) would provide a more balanced understanding. Finally, investigating alternative mediators (e.g., trust, identification) and moderators (e.g., organizational support) would further enrich the theoretical model.

## Data Availability

The original contributions presented in the study are included in the article/supplementary material, further inquiries can be directed to the corresponding author.
